# Factors associated with retention on medications for opioid use disorder among a cohort of adults seeking treatment in the community

**DOI:** 10.1186/s13722-022-00299-1

**Published:** 2022-03-07

**Authors:** Breanne E. Biondi, Brent Vander Wyk, Esther F. Schlossberg, Albert Shaw, Sandra A. Springer

**Affiliations:** 1 Department of Health Law, Policy and Management, Boston University School of Public Health, Boston, MA USA; 2 Department of Internal Medicine, Section of Geriatrics, Yale School of Medicine, New Haven, CT USA; 3 Department of Internal Medicine, Section of Infectious Diseases, Yale School of Medicine, Yale AIDS Program, New Haven, CT USA; 4 Center for Interdisciplinary Research On AIDS, Yale University School of Public Health, New Haven, CT USA; 5 Department of Internal Medicine, Section of Infectious Disease, Yale AIDS Program, 135 College Street, Suite 323, New Haven, CT 06510 USA

**Keywords:** Medications for opioid use disorder, Methadone, Buprenorphine, Opioids, Retention

## Abstract

**Background:**

Medication treatment for opioid use disorder (OUD) (MOUD; buprenorphine and methadone) reduces opioid use and overdose. Discontinuation of MOUD can quickly lead to relapse, overdose and death. Few persons who initiate MOUD are retained on treatment, thus it is critical to identify factors associated with retention.

**Methods:**

Evaluated data was from an ongoing prospective cohort study of adults aged 18 or older with DSM-5 moderate to severe OUD seeking MOUD in the community and followed for 6 months. Participants were considered retained on MOUD through 6 months if they reported taking MOUD at every study interview without discontinuation. A high dose of MOUD was defined as a methadone dose > 85 mg or buprenorphine dose ≥ 16 mg. Multivariable logistic regression was conducted to assess factors associated with 6-month MOUD retention.

**Results:**

A total of 118 participants (73% male, 58% white, 36% with HIV) were included. Buprenorphine was initiated by 58% and 42% started methadone. MOUD retention was 49% and 58% among buprenorphine and methadone, respectively, at 6-months. In adjusted models, a high MOUD dose (OR = 4.71, 95% CI 2.05–10.84) and higher pain interference (OR = 1.59, 95% CI 1.15–2.19) was associated with MOUD retention.

**Conclusions:**

Adequate dosing of MOUD leads to improved retention on MOUD. Further, persons with high pain interference at baseline had higher odds of retention on MOUD. Both methadone and buprenorphine have analgesic effects, thus those with high pain interference could have dual benefits of MOUD for treating OUD and pain. Interventions should be tailored to improve adequate MOUD dosing to improve retention on MOUD.

## Background

The United States is currently experiencing a nationwide public health crisis, the opioid epidemic [1]. This is now mainly due to illicitly manufactured fentanyl that is causing an increasing number of opioid overdose deaths across the country [2]. From 2005 to 2019, there was a nationwide increase of opioid-related hospitalizations and From April 2020 to 2021 over 100,000 Americans died due to opioid overdoses [3, 4].

The most effective way to treat opioid use disorder (OUD) is with one of three FDA approved medications: methadone, buprenorphine, or extended release naltrexone [5]. Medications for opioid use disorder (MOUD) are effective at reducing opioid cravings, relapse and overdose [6]. Additionally, they have the added benefit of improving health and physical functioning, reducing sexual and injection drug use (IDU) HIV risk behaviors, and reducing transmission of infections such as HIV and Hepatitis C virus [7–10]. These benefits occur when persons with OUD remain on MOUD, however around half of persons started on MOUD remain retained on treatment through 12 months [11], although reported ranges vary as wide as 26–91% [12].

There are demographic, clinical, and social factors that affect retention in MOUD treatment, and thus return to opioid use. Some of these factors have been studied more extensively than others, for example, those who are older have better retention on MOUD [11]. Other factors that have been identified to improve retention include stable housing, adequate or higher doses of methadone or buprenorphine, and reductions and cessation in other drug use [13–15]. Additionally, it has been reported that persons taking methadone often have better retention compared to those taking buprenorphine [11]. Stimulant use, including cocaine and methamphetamine, has been associated with decreased MOUD retention [16].

Some factors, however, are less studied or have mixed results, such as gender, where some studies report no effect, and other studies report that men have lower retention rates compared to women [17]. Additionally, mental health diagnoses and physical health status have demonstrated mixed results regarding their effects on MOUD retention [11]. Further, improvements in retention on MOUD have been shown to improve quality of life (QoL), [18] however, few studies examine if QoL can influence MOUD retention. Transportation as defined as motor vehicle services have also been associated with increased retention on MOUD, while use of transportation vouchers or bus passes have not shown an effect on MOUD retention [19]. However, studies evaluating client satisfaction with transportation services, regardless of type of services, have not been conducted. Finally, adults with OUD who seek MOUD after release from carceral settings may have decreased MOUD retention compared to those who initiate MOUD in the community due to barriers affecting recently released persons such as access to insurance, housing difficulties and low social support.

MOUD retention measured through six months is a quality measure endorsed by the National Quality Forum (NQF), the gold standards for healthcare measurements [20]. We therefore sought to examine factors associated with MOUD retention to understand if there are factors that could be influenced to improve OUD treatment and associated outcomes. In this analysis, retention on MOUD through 6 months was analyzed among a cohort of adults with OUD actively seeking methadone and buprenorphine treatment in the community.

## Methods

### Data and sample

We evaluated data from a prospective cohort study of persons with Diagnostic and Statistical Manual of Mental Disorders (DSM-5) diagnosed moderate to severe OUD living with and without HIV who were initiating treatment with methadone or buprenorphine in the community [21]. Participants starting MOUD at an approved study site were screened on-site and enrolled the day they were to begin MOUD. Details of the study protocol were previously published [21]. After informed consent was obtained, participants underwent the baseline interview assessment prior to starting MOUD and then had follow-up interviews at day 7, day 14, and months 1, 3, and 6 after MOUD initiation [21]. For this analysis we excluded 2 participants; one participant died before study completion and the other did not complete their month 6 interview. We only evaluated participants who had data through 6 months or who reported stopping MOUD before 6 months. In the parent cohort study, participants were still followed and underwent follow-up interviews even if they stopped treatment with MOUD.

### Primary outcome

The primary outcome for this analysis was retention on MOUD through 6 months, as this is an NQF quality measure. Participants were considered retained on MOUD through 6 months if they reported taking MOUD at every study interview and did not report stopping taking MOUD at any follow up visit (MOUD was confirmed with treatment clinics and through self-report). Participants could self-report missing doses and still be considered retained on MOUD if they were still receiving prescribed treatment through confirmation with their clinicians and if they had a positive urinalysis for the MOUD they were prescribed. Participants who changed medications (i.e., from methadone to buprenorphine), were considered retained if they did not stop treatment before changing medication.

### Primary exposures

In this exploratory analysis, we focused on exposures identified in the literature to be associated with MOUD retention. The following binary exposures were assessed: housing status (houseless), [16] HIV status, [11] cocaine use [11] (dichotomized as ≥ 1 day per week compared to < 1 day a week or no use, in order to assess current, sustained cocaine use), DSM-5 diagnosed depression [11] (via the Mini International Neuropsychiatric Interview (MINI) v7.0.2) [22], referral to treatment from prison/jail, [16] employment status, [11] and a high dose of MOUD [12] at any time during the study period. A high dose of MOUD was defined as a methadone dose > 85 mg or buprenorphine dose ≥ 16 mg (based on the systematic review conducted by Mattick et al. [23]) Since the cohort included both those receiving methadone and buprenorphine, this variable was dichotomized into ‘high dose’ of MOUD and ‘not a high dose’ of MOUD.

Continuous and ordinal exposures included quality of life (QoL) scores (physical, psychological, social relationships, and environment) from the WHOQoL BREF [24] (scores range from 0 to 100), to examine if baseline QoL scores are associated with retention. OoL was examined to see if baseline QoL is associated with retention, rather than the reverse (retention on MOUD leading to better QoL), that has been examined in other published studies [18]. We also aimed to evaluate what aspects of QoL are associated with MOUD retention. Other exposures included opioid craving (how much participants are currently craving opioids, (Likert scale from 1 to 10, with 10 indicating the most craving), satisfaction with transportation (Likert scale from 1 = very dissatisfied to 5 = very satisfied), and pain interference (to what extent do you feel that physical pain prevents you from doing what you need to do?).

### Covariates

The following covariates were used in adjusted models: participant age (in years), gender, treatment type (methadone or buprenorphine), and baseline opioid use severity score [from the Alcohol, Smoking and Substance Involvement Screening Test (ASSIST)] [25].

### Statistical analysis

#### Unadjusted estimates

We describe the number and percent of categorical variables and the means and standard deviations for continuous variables in Table 1. A series of bivariate analyses were run to test the relationship of targeted explanatory variables and MOUD treatment retention. Each variable was entered in a logistic regression with retention (successful retention = 1) as the outcome variable. All models included an intercept term. Significance for each variable was assessed using a Wald’s χ^2^test. Uncorrected and false discovery rate (FDR) corrected p values are reported. FDR is used when multiple comparisons are conducted to reduce the chance of Type I error.

**Table 1:** Demographic and baseline characteristics

Variable	Overall N = 118	Buprenorphine N = 68	Methadone N = 50
Gender—N (%)			
Woman	32 (27.12%)	21 (30.88%)	11 (22.00%)
Man	86 (72.88%)	47 (69.12%)	39 (78.00%)
Race N (%)			
White	69 (58.47%)	36 (52.94%)	33 (66.00%)
Black/African American	29 (24.58%)	19 (27.94%)	10 (20.00%)
Other	10 (8.47%)	6 (8.82%)	4 (8.00%)
More than one race	6 (5.08%)	5 (7.35%)	1 (2.00%)
Native American	4 (3.39%)	2 (2.94%)	2 (4.00%)
Hispanic or Latinx N (%)	37 (31.36%)	27 (39.71%)	10 (20.00%)
Age—mean (SD)	42.41 (12.64)	44.12 (12.04)	40.08 (13.19)
Education—HS equivalent or greater—N (%)	93 (78.81%)	51 (75.00%)	42 (84.00%)
Pain interference—mean (SD)	2.55 (1.28)	2.72 (1.35)	2.32 (1.15)
Transportation satisfaction—mean (SD)	3.12 (1.27)	3.03 (1.27)	3.24 (1.27)
High dose of MOUD^a^—N (%)	59 (50.00%)	38 (55.88%)	21 (42.00%)
Six-month MOUD retention—N (%)	62 (52.54%)	33 (48.53%)	29 (58.00%)
Houseless—N (%)	38 (32.20%)	25 (36.76%)	13 (26.00%)
Working in past 30 days—N (%)	24 (20.34%)	15 (22.06%)	9 (18.00%)
Insurance coverage in prior 30 days—N (%)	116 (98.31%)	68 (100.00%)	48 (96.00%)
Referred from carceral setting (prison or jail)	19 (16.10%)	10 (14.71%)	9 (18.00%)
Living with HIV—N (%)	43 (36.44%)	32 (47.06%)	11 (22.00%)
MINI Major Depressive Disorder, current—N (%)	31 (28.18%)	18 (27.69%)	13 (28.89%)
MINI Major Depressive Disorder, past—N (%)	45 (40.91%)	27 (41.54%)	18 (40.00%)
WHOQoL BREF Physical—mean (SD)	13.09 (3.59)	13.04 (3.73)	13.15 (3.44)
WHOQoL BREF Psychological—mean (SD)	13.42 (3.34)	13.61 (3.30)	13.18 (3.41)
WHOQoL BREF Social Relationships—mean (SD)	13.38 (3.77)	13.53 (3.81)	13.17 (3.74)
WHOQoL BREF Environment—mean (SD)	13.30 (2.88)	13.10 (2.82)	13.57 (2.97)
Opioid Craving Scale—Mean (SD)	6.85 (3.47)	6.41 (3.72)	7.44 (3.02)
ASSIST Any Cocaine use, past 3 months—N (%)			
No	63 (53.39%)	40 (58.82%)	23 (46.00%)
Yes	55 (46.61%)	28 (41.18%)	27 (54.00%)

*MOUD *medications for opioid use disorder; *MINI *Mini International Neuropsychiatric Interview; *WHOQoL* *BREF *World Health Organization Quality of Life, 26 item version; *ASSIST *Smoking and Substance Involvement Screening Test

^a^High dose of MOUD defined as methadone dose > 85 mg or buprenorphine dose ≥ 16 mg:

#### Adjusted estimates

A subset of variables was each individually entered into a multivariable logistic regression with retention (successful retention = 1) as the outcome variable. Variables included those that were significant in unadjusted analyses that were associated with MOUD retention. Covariates were included in these models. The significance of each variable of interest was assessed using a Wald test, and overall model goodness-of-fit was assessed with the Hosmer–Lemeshow test. Odds ratios and 95% CI are reported, as well as the marginal probabilities of treatment retention for significant variables. Marginal (predicted) probabilities are reported given that odds ratios overestimate risk when the prevalence of an outcome is high, and because the magnitude of ORs are scaled by an arbitrary factor [26]. The marginal probabilities are the probability of MOUD retention for each variable, holding all other variables constant.

## Results

A total of 118 participants were included, and were mostly male (73%) and white (58%). Ninety-four had completed a month 6 interview. Nearly a quarter of participants were Black/African American, and 31% were Hispanic/Latinx (see Table 1 for baseline characteristics). Buprenorphine was initiated by 68 (58%) of participants, and methadone in 50 (42%) participants. Over a third of the cohort had HIV (N = 43, 36.4%, Table 1). Participants with HIV were more likely to start buprenorphine (N = 32, 74.4%) compared to those without HIV (N = 36, 48.0%). At 6 months, 53% were retained on MOUD (49% of those who started buprenorphine and 58% of those who started methadone were retained through 6 months). Nearly all participants (98%) had health insurance coverage at baseline.

### Unadjusted estimates

The point estimate and 95% CI for the unadjusted odds ratio for each variable is presented in Table 2. Considering uncorrected tests, the variable indicating a high dose at any time (reference value = 0; never a high dose) significantly increased the odds of retention (p < 0.001). Similarly, increases in Pain Interference Level also significantly increased the odds of retention (p < 0.05). Being houseless (reference value = 0; having a place to stay) was associated with lower odds of retention (p = 0.052). When correcting for multiple comparisons using a FDR (q < 0.05,) only high dose of MOUD was statistically significant.

**Table 2:** Bivariate unadjusted associations with MOUD retention

Variable	Odds Ratio	95% CI		P-value (from Wald’s χ^2^test)
Age	1.00	0.97	1.03	0.95
Gender	0.48	0.21	1.11	0.09
MINI Major Depressive Disorder, Current or Past	1.04	0.48	2.24	0.92
High dose of MOUD^a^	4.12	1.91	8.89	0.00
Frequency of cocaine use	0.95	0.40	2.25	0.91
Houseless	0.46	0.21	1.01	0.05
Opioid Craving Score	0.96	0.86	1.07	0.44
WHOQoL BREF physical	0.95	0.85	1.05	0.29
WHOQoL BREF psychological	1.02	0.92	1.14	0.67
WHOQoL BREF—social relationships	1.09	0.99	1.20	0.09
WHOQoL BREF—environment	1.11	0.98	1.27	0.11
HIV status	0.92	0.43	1.94	0.82
Satisfaction with transportation	1.13	0.85	1.50	0.41
Pain interference	1.44	1.07	1.94	0.02
Referred to treatment by prison/jail	1.00	0.38	2.68	0.99
Employed	0.88	0.36	2.16	0.78

*MOUD *medications for opioid use disorder; *MINI *Mini International Neuropsychiatric Interview; *WHOQoL* *BREF *World Health Organization Quality of Life, 26 item version

^a^High dose of MOUD defined as methadone dose > 85 mg or buprenorphine dose ≥ 16 mg

### Adjusted estimates

Point estimates and 95% CI for the adjusted odds ratio for each variable of interest is presented in Table 3. All Homer-Lemeshow tests of goodness-of-fits were insignificant (p > 0 0.05), indicating that all models provided a good fit to the data. Adjusting for age, gender, MOUD treatment type, and baseline injectable opioid use score did not appreciably change the relationship of each variable to retention on treatment. Additionally, MOUD type (buprenorphine/methadone) was not significant in the model. A high dose of MOUD (OR = 4.71, 95% CI 2.05–10.84) and higher pain interference (OR = 1.59, 95% CI 1.15–2.19) were both associated with MOUD retention. Being houseless was associated (but not statistically significant) with lower odds of MOUD retention (OR = 0.44, 95% CI 0.20–1.00).

**Table 3:** Regression results of factors associated with MOUD retention

Variable	Predicted probability (95% CI)^b^	Odds ratios (95% CI)
Not houseless	0.59 (0.48–0.69)	
Houseless	0.40 (0.24–0.55)	0.44 (0.20–1.00)
Not on a high dose of MOUD	0.35 (0.23–0.47)	
On a high dose of MOUD^a^	0.70 (0.58–0.82)	4.71 (2.05–10.84)
Pain interference (to what extent do you feel that physical pain prevents you from doing what you need to do)?		1.59 (1.15–2.19)
Not at all	0.36 (0.23–0.50)	
A little	0.47 (0.37–0.56)	
A moderate amount	0.57 (0.48–0.67)	
Very much	0.67 (0.55–0.80)	
An extreme amount	0.76 (0.61–0.91)	

^a^High dose of MOUD defined as methadone dose > 85 mg or buprenorphine dose ≥ 16 mg. Control variables in the model included baseline opioid use severity score, age, gender, and type of MOUD (methadone or buprenorphine)

^b^Predicted probabilities measure the probability of MOUD retention for each variable while holding other variables in the model constant

Being housed increased the probability of MOUD retention by 19 percentage points, and receiving a high dose of MOUD at any time point increased the probability of MOUD retention by 35 percentage-points. The probability of being retained on MOUD increases with increasing pain interference (Table 3).

## Discussion

In this on-going current cohort study of adults with OUD seeking treatment with medication treatment for OUD (MOUD) in the community, we found that adequate dosing of MOUD leads to improved retention on MOUD. This result is consistent with previous findings demonstrating that higher methadone and buprenorphine doses are associated with increased retention in treatment [11, 27], while our findings are unique as they are from a real-world on-going cohort of adults seeking MOUD in the community with a high proportion of individuals living with HIV. Additionally, this study is important as a quarter of participants were Black/African American and over 30% were Hispanic/Latinx, a strength given that minorities are typically underrepresented in medication trials for OUD and improving diversity in inclusion in these studies is important [28].

There are many barriers that persons with OUD have to overcome to not only initiate MOUD, but also to receive adequate dosing of MOUD in the community. Regulations include dispensing at federally regulated opioid treatment programs (OTPs), daily visits to the OTP to receive methadone at the beginning of treatment [29], and strict rules regarding missed doses and use of drugs [30]. During the COVID-19 pandemic, OTPs were allowed to provide up to 28 days of take-home methadone doses to patients, greatly reducing barriers. During these COVID-19 pandemic-related changes to OTP prescribing, there were no changes in positive urine drug tests for methadone [31]. Allowing these COVID-19 pandemic-related changes to continue long term may help patients stay retained on methadone with adequate dosing [30]. Additionally, there is evidence that patient participation in methadone dose decisions leads to better perceptions of dose adequacy [32, 33], and patient-centered models for office-based buprenorphine are preferred by patients [34]. Future work should focus on including patient perceptions in methadone and buprenorphine dosing to determine if this would improve retention in treatment.

While our study did not find a statistically significant effect of housing on MOUD retention, there was a strong association between lack of housing and reduced retention on MOUD. Lack of housing remains a significant barrier to accessing treatment and MOUD retention. Individuals without housing often have competing priorities, including finding shelters to stay in, employment, and food. Housing First programs, which prioritize providing housing for unhoused individuals, followed by support services have positive benefits [35, 36]. These include increased housing stability, reductions in criminal justice involvement, and better quality of life [35]. Evidence varies on the effect of Housing First on substance use outcomes, with some studies showing increased MOUD adherence while others show decreased adherence [35–37]. More research is needed to evaluate if housing first could improve retention on MOUD and reduction in opioid overdoses and other negative consequences of opioid relapse.

Persons with high pain interference at baseline had higher odds of retention on MOUD in our study. Both methadone and buprenorphine have analgesic effects, thus those with high pain interference could have dual benefits of MOUD for treating OUD and pain. In one study, over half of participants noted that chronic pain was a reason for maintaining their methadone dose, as it provided comfort from pain [33].

In this study, we did not find any association with gender or mental health and MOUD retention, which has demonstrated mixed results from previously published studies [11, 17]. This could be due to our small sample size combined with less than 30% of the sample being women. Additionally, for mental health assessment we only used baseline measures. Mental health status may have heterogenous effects, and may present a challenge for retention in treatment or help with retention when mental health treatment is combined with substance use treatment [38].

The factors identified in this study, as well as others in the literature can help substance use treatment service programs and clinicians in retaining people in MOUD treatment. First, higher dose is an important factor to consider, and we recommend patient participation in dosing decisions. Second, identifying patients’ pain management needs along with OUD treatment can help patients stay retained on treatment. Addressing housing issues are also important but may be difficult for programs and clinicians to address, depending on existing community resources. Broader policy changes may better address this issue, and clinicians and researchers should advocate for affordable housing and federal funding for homelessness programs [39].

This analysis has some limitations; first, our sample size is small which likely impacted the results. This analysis is a secondary analysis of a study aimed to generate hypotheses about the biological effects of MOUD treatment on persons with HIV and without HIV [21]. Because of the exploratory nature of this study, we accounted for multiple comparisons in our unadjusted analyses. This limited the number of significant differences we were able to report for MOUD retention, but reduced our chances of making any type I errors. However, because of the exploratory nature of this analysis, further studies that can determine causality between these factors and MOUD retention are needed. Individuals in this cohort were all choosing to start MOUD treatment in the community, and thus may be more representative of participants engaging in treatment compared to those enrolled in randomized controlled trials. Additionally, because we only included those with 6 months of study follow-up, selection bias could impact these results.

## Conclusion

This study adds to the existing literature on identifying factors associated with MOUD retention. Importantly, higher dose of buprenorphine and methadone improved retention as has been shown in other published research [11]. Through improving retention on MOUD, reductions in relapse to opioid use, overdose deaths and HIV and HCV transmission could result. More research is needed to understand and improve provider decisions on form and dose of MOUD, inclusion of patient preferences and pain interference, as well as improvements of housing and other structural determinations of healthcare to improve retention on medication treatment for OUD.

## Data Availability

Datasets used and/or analyzed during the systematic review are available from the corresponding author on reasonable request.

## Abbreviations

OUD: Opioid use disorder
MOUD: Medication treatment for opioid use disorder
IDU: Injection drug use
NQF: National Quality Forum
NIDA: National Institute of Drug Abuse
DSM-5: Diagnostic and Statistical Manual of Mental Disorders
WHOQoL BREF: World Health Organization Quality of Life Assessment—Brief
MINI: Mini International Neuropsychiatric Interview
ASSIST: The Alcohol, Smoking and Substance Involvement Screening Test
FDR: False discovery rate
